# Fatal Monkeypox in Wild-Living Sooty Mangabey, Côte d’Ivoire, 2012

**DOI:** 10.3201/eid2006.131329

**Published:** 2014-06

**Authors:** Aleksandar Radonić, Sonja Metzger, Piotr Wojtek Dabrowski, Emmanuel Couacy-Hymann, Livia Schuenadel, Andreas Kurth, Kerstin Mätz-Rensing, Christophe Boesch, Fabian H. Leendertz, Andreas Nitsche

**Affiliations:** Robert Koch Institute, Berlin, Germany (A. Radonić, S. Metzger, P.W. Dabrowski, L. Schuenadel, A. Kurth, F.H. Leendertz, A. Nitsche);; Laboratoire de la Pathologie Animale, Bingerville, Côte d’Ivoire (E. Couacy-Hymann);; Deutsches Primatenzentrum, Göttingen, Germany (K. Mätz-Rensing);; Max-Planck-Institute for Evolutionary Anthropology, Leipzig, Germany (C. Boesch)

**Keywords:** Poxvirus, monkeypox virus, zoonoses, Taï National Park, viruses, Côte d’Ivoire

## Abstract

We isolated a monkeypox virus from a wild-living monkey, a sooty mangabey, found dead in Taï National Park, Côte d’Ivoire, in March 2012. The whole-genome sequence obtained from this isolate and directly from clinical specimens showed its close relationship to monkeypox viruses from Western Africa.

Among the poxviruses are several species of orthopoxviruses (OPVs) that are pathogenic to humans, including monkeypox virus (MPXV) and variola virus (VARV). MPXV was first discovered in laboratory captive monkeys in Copenhagen in 1958 (*1*). After the eradication of VARV during the 1970s, MPXV became the highest pathogenic OPV infection in humans. On the basis of epidemiologic and sequence data, strains of MPXV can be assigned to a West African or a Congo Basin clade; viruses from the Congo Basin clade show more pronounced illness, death, viremia, and human-to-human transmission than do strains from the West African clade (*2*,*3*).

Serologic studies showed that monkeys from Africa have OPV antibodies, but no natural case of MPXV has been reported in wild-living primates (*4*,*5*). The only MPXV isolate obtained from an animal in the wild was from a Thomas’s rope squirrel (*Funisciurus anerythrus*) caught in Democratic Republic of the Congo in 1985 (*6*). Here we describe natural MPXV infection in a sooty mangabey (*Cercocebus atys*) found dead in Taï National Park (TNP), Côte d’Ivoire.

## The Study

During a long-term program to monitor deaths in wildlife, an infant mangabey was found dead in the TNP in March 2012. The body did not show any apparent injuries, and the animal had died relatively recently, as indicated by the presence of blowfly eggs but absence of maggots. Multiple skin lesions typical of MPXV infection occurred as dark red crusts 5–7 mm in diameter, partly confluent, which were disseminated over the body. Extremities were mainly affected; fewer lesions were seen on the belly and none on the back.

A full necropsy was performed under high-level safety measures, and samples of all organs and blood were collected and preserved in liquid nitrogen and 10% buffered formalin (*7*). Histologic analysis of the skin showed eosinophilic inclusion bodies, suggesting that an OPV infection had caused the ulcers. Severe bacterial secondary infection of the ulcers also was observed, as well as bacteremia that might have contributed to the pathologic changes and death.

DNA was extracted from different tissues, and quantitative PCR for OPV DNA (rpo18) and a cellular target (c-myc) were performed as described (*8*). We found a high viral DNA load in relation to cellular DNA in all tissues, except muscle, indicating a systemic infection, with particularly high loads in a skin lesion and from a throat swab sample (Table). An immunofluorescence assay performed on MPXV-infected cells showed titers of 320 for IgG and 80 for IgM, indicating an acute OPV infection.

**Table:** Results of quantitative PCR from tissues of a wild-living sooty mangabey (*Cercocebus atys*), Taï National Park, Côte d’Ivoire, March 2012*

Tissue	C_t_ OPV rpo18	C_t_ c-myc	ΔC_t_, rpo18–c-myc
Spleen	32.0	22.1	–9.9
Lung	34.3	27.8	–6.5
Kidney	28.2	18.9	–9.3†
Skin	16.9	26.9	9.9
Liver	30.3	20.0	–10.3†
Heart	32.2	25.0	–7.2
Intestine	32.2	31.1	0.1
Muscle	ND	ND	ND
Thymus	15.8	19.7	3.9†
Throat swab	21.5	28.4	6.9†
Lymph node	24.1	20.2	–3.9†

*Virus DNA (rpo18) was quantified in relation to cellular c-myc DNA in 1 μL DNA; higher values indicate higher virus loads in a respective tissue. Ct, cycle threshold; OPV, orthopoxvirus; ND, OPV DNA not detectable. †5 μL used in PCR.

Virus from skin tissue was propagated in HEp-2 cells, infected cells were harvested, and DNA was extracted. We performed library preparation and sequencing on an Ion Torrent PGM with an Ion PGM Sequencing 200 Kit (Life Technologies, Darmstadt, Germany) (average read length = 93 bases). Sequences were analyzed by using Geneious (Biomatters, Auckland, NZ). At first, a de novo assembly from PGM fastq-data was conducted, followed by mapping of the resulting contigs to all 11 MPXV genomes published in the National Center for Biotechnology Information to obtain orientation and to form a consensus sequence. The reads were mapped again to the consensus sequences to identify assembly errors. These efforts resulted in a single 197,571-bp genome. Additionally, DNA preparations from the mangabey’s skin and throat swab specimens were subjected directly to an Illumina HiSeq 1500 (San Diego, CA, USA), sequencing 150 + 150 bases (paired end). The genome could also be assembled to 190,562 bp by using Illumina data only (2.9% of the 285 million Illumina reads could be used for the MPXV assembly, compared with 45% of the 3.5 million reads from the PGM), indicating that the viral genome can be assembled by sequencing specimens of high viral load directly without previous virus propagation.

Finally, we obtained a 12,395-fold coverage of the MPXV genome from Illumina data and a 732-fold coverage for data from the PGM. Eighty-four percent of the reads from the skin sample could be mapped to *Staphylococcus aureus*. These data provide evidence of the usability of direct Illumina sequencing for metagenomic analysis. Combining gained sequence data showed a full genome of MPXV-TNP with 200,035 bp (GenBank accession no. KJ136820).

**Figure 1:**
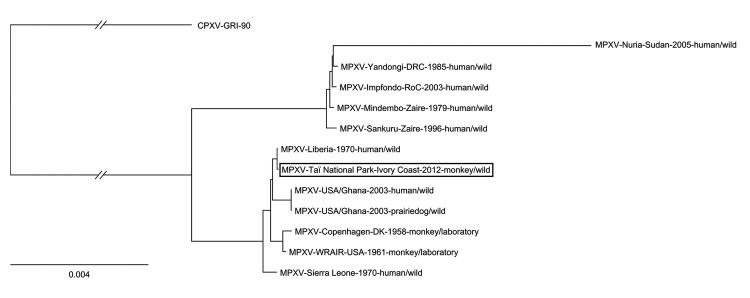
Phylogenetic position of the MPXV-TNP isolate (framed in green) from a wild-living sooty mangabey (*Cercocebus atys*), March 2012, within the West African clade. MPXV-TNP is closely related to the strain isolated from a human in Liberia in 1970. Calculated with MrBayes (with gaps) as binary model (http://mrbayes.csit.fsu.edu). MPXV, monkeypox virus; TNP, Taï National Park (Côte d’Ivoire). Scale bar indicates nucleotide substitutions per site.

**Figure 2:**
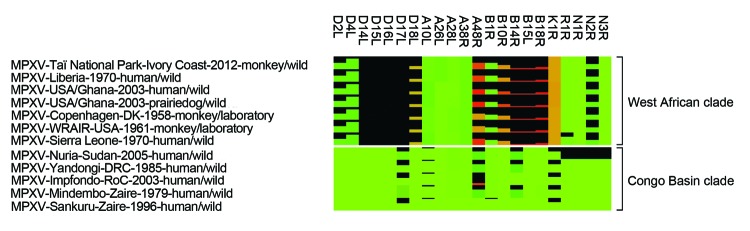
Heat map of MPXV proteins with rather low conservation. Shown is the comparison of protein length and identity. The degree of protein truncation is represented as a black bar. The differences in protein identity of the remainder of the proteins are represented by color gradation ranging from green (100% protein identity) to brown (≈50% protein identity) to red (0% protein identity). Only proteins with protein length or identity <95% are shown. Protein names are based on MPXV-Sankuru-Zaire-1996. MPXV, monkeypox virus.

All known MPXV genomes, including MPXV-TNP, and the genome of cowpox virus GRI-90 (used as outgroup) were truncated to include the sequence information between the first and last coding region for further phylogenetic analysis (MrBayes, v. 3.2.1 (http://mrbayes.sourceforge.net/), with gaps as binary model). MPXV-TNP is closely related to a human MPXV isolate detected in the neighboring country of Liberia in 1970 (Figure 1). Together with other isolates from this geographic region, MPXV-TNP belongs to the West African clade of MPXV (*2*,*9*). Additionally, protein sequences of MPXV-TNP were more similar to those of sequences from the West African clade than to those of the Congo Basin clade (Figure 2).

Sequence comparison of West African and Congo Basin MPXVs by other researchers showed individual genes that are conserved across the 2 clades and are speculated to be responsible for the different pathogenicity of the viruses (*2*,*3*). For example, we analyzed the sequence of the immunomodulatory protein IL-1β receptor (B14R), which moderated the severity of vaccinia virus infection in a mouse model (*10*). MPXV-TNP encodes a further truncated sequence variant of the IL-1β protein (confirmed by Sanger sequencing) that is unique among known MPXV strains. Unfortunately, the effects of IL-1β receptor fragmentation on functionality and on virulence of MPXV is not known (*11*).

## Conclusions

The case described here suggests that mangabeys can be fatally infected with MPXV in nature and have high viral loads found in various tissues. Phylogenetic analysis showed that the newly identified MPXV-TNP strain is closely related to MPXV isolated from humans in Liberia and Sierra Leone in 1970 and can be assigned to the less virulent West African clade of MPXV, which seems surprising. A possible explanation is that MPXV disease progression appears to be rare in wild-living monkeys, and this case was the first observed in both regions: Central Africa, with more lethal strains, and West Africa, with less lethal strains. Also, the mangabey was only a few weeks of age and might not have had a fully developed immune system, and this factor and its secondary bacterial infection were likely to have contributed to disease severity. Moreover, individual immunologic defects cannot be ruled out. Nevertheless, very few monkeys are under systematic observation by humans, and infection of humans with the MPXV-TNP strain cannot be ruled out, even though no cases in humans were recognized in this area during the same period.

This case demonstrates that wild primates can serve as indicators for specific pathogens in certain regions (*12*). The local human population hunts and eats potential reservoir species, such as rodents, and therefore follow-up investigations of human infections and the prevalence of MPXV in reservoir species are needed to pinpoint the zoonotic risk posed by MPXV in the area. Additionally, studying the role of MPXV infections in wild nonhuman primates could enhance understanding of the natural history of this virus.
